# Reviewer acknowledgments

**DOI:** 10.1002/mlf2.70069

**Published:** 2026-02-09

mLife thanks the following reviewers for their great support and contribution in 2025:

Tianchen An

Pascal Arnoux

Rohan Balakrishnan

Haluk Beyenal

Arun K. Bhunia

Haimanti Biswas

Eric Boyd

Colin Buttimer

Shang Cai

Jodi L. Camberg

Liu Cao

Pengbo Cao

Eric Caragata

Jieliang Chen

Yuxin Chen

Maria Chuvochina

Thomas A. Clarke

Cunha Violette Da

Lei Dai

Ajai A. Dandekar

Yunkun Dang

Vijaya Kumar Deekshit

Ling Deng

Xin Deng

Yinyue Deng

Muhe Diao

Binbin Ding

Huijuan Dong

Tao Dong

Wei‐Liang Dong

Shishen Du

Chengguo Duan

Yuange Duan

Shigeki Ehira

Jie Feng

Xiaoyuan Feng

Xu Feng

Yue Feng

Paulo Igor Firmino

Greg Fournier

Chuanhai Fu

Tian‐Min Fu

Yang Fu

Danny Fung

Cheng Gao

Haichun Gao

Yongqiang Gao

Zheng Gao

Rodolfo Garcia‐Contreras

Xue Guo

Yunxue Guo

Martin Hagemann

Guanzhu Han

Wenyuan Han

Yuling Han

Thomas Hanscheid

Ji‐Zheng He

Ya‐Wen He

Aurelia Hiron

Brian Ho

Mengfei Ho

Dawn Holmes

Yoichi Honda

Shengwei Hou

Chunyi Hu

Fupin Hu

Hongbo Hu

Jiafen Hu

Jiao Hu

Jinhu Huang

Zhiwei Huang

Yixin Huo

Yamini Jangir

Ning Jia

Tianyuan Jia

Zhongjun Jia

Huahua Jian

Daohong Jiang

Hongmei Jing

Yaming Jiu

Hakuto Kageyama

Chikara Kaito

Sarkar M. A. Kawsar

Weidong Kong

Naoki Konno

Tomoko Kubori

Amel Latif

Maggie Lau Vetter

Houkai Li

Ming Li

Rong Li

Yan Li

Yifang Li

Yong Li

Zhiyuan Li

Guodong Liang

Jun Lin

Wei Lin

Steven Lindow

Chao Liu

Chang Liu

Cuihua Liu

Gao‐Qiang Liu

Hongwei Liu

Huiquan Liu

Jianming Liu

Ningning Liu

Pengfei Liu

Xue Liu

Yang Liu

Yongqin Liu

Zhao‐Qing Luo

Jitao Lv

Liping Ma

Yingfei Ma

Zhe Ma

Yuxin Mao

Joseph Marquardt

Carolina Martinez

Tom A. Mendum

Randy M. Morgenstein

Hirotada Mori

Da‐Shuai Mu

Conrad W. Mullineaux

Ryuhei Nakaruma

Yong Nie

Kang Ning

Siqiang Niu

Julie D. Pourtois

Bao‐Sheng Qiu

Dongru Qiu

Giordano Rampioni

Yang‐Zhi Rao

Christian Rinke

Muhammad Bilal Sadiq

Roberto Kopke Salinas

Miguel Semedo

Konstantin Severinov

Kathy Ho Yen Shair

Chunmin Shan

Xiaolong Shao

Zongze Shao

Feng Shen

Xihui Shen

Yulong Shen

Yu Shi

Francesco Smedile

Yi Song

Yu Song

Hongwei Su

Clifford Swanson

Yu Tang

Liang Tao

Pan Tao

Xiaoyuan Tao

Xuanyu Tao

Regina Verena Taudte

Chang‐Fu Tian

Areg A. Totolian

Pier‐Luc Tremblay

Qichao Tu

Schaik Erin Van

Bo Wang

Chengshu Wang

Decheng Wang

Fang‐Fang Wang

Fengping Wang

Jianjun Wang

Jue Wang

Qiang Wang

Rui Wang

Weishan Wang

Yinzhao Wang

Yongcheng Wang

Hai‐Lei Wei

Michael Wells

William Whitman

Xiong Wu

Weihui Wu

Yang Wu

Wei Xia

Yu Xia

Genhui Xiao

Lihua Xiao

Jianping Xie

Wei‐Yue Xing

Jinling Xu

Ying Xu

Guanhua Xuan

Aixin Yan

Huilin Yan

Zhen Yan

Chunfu Yang

Jian Yang

Jian Yang

Liang Yang

Qiwen Yang

Sidi Yang

Xinxing Yang

Yuyi Yang

Lingchong You

Hanqing Yu

Pingfeng Yu

Qinglu Zeng

Cheng‐Cai Zhang

Heng Zhang

Ju‐Yuan Zhang

Li Zhang

Qijing Zhang

Rui Zhang

Shouyue Zhang

Yu Zhang

Jinxin Zhao

Wei Zhao

Youbao Zhao

Xiangkai Zhen

Zhai Zhiwen

Wei Zhou

Jianuan Zhou

Tian Zhou

Junhao Zhu

Kui Zhu

Yan Zhu

Yibin Zhu

